# Prevalence of mental health and behaviour problems among adolescents in the English-speaking Caribbean: systematic review and meta-analysis

**DOI:** 10.1007/s44192-023-00037-2

**Published:** 2023-05-18

**Authors:** Shaun Liverpool, Jamal Prescod, Brent Pereira, Catherine Trotman

**Affiliations:** 1 Faculty of Health, Social Care and Medicine, Edge Hill University, Ormskirk, UK; 2 Evidence Based Practice Unit, Anna Freud National Centre for Children and Families, London, UK; 3 Faculty of Social Sciences, The University of the West Indies, Cave Hill, St Michael, Barbados; 4 Department of Counselor Education, The Chicago School of Professional Psychology, Chicago, USA

**Keywords:** Mental health, Prevalence, Children, Adolescents, Young people

## Abstract

**Objective:**

Children and young people (CYP) from low-and-middle-income and developing countries are at risk of poor mental health and wellbeing. Yet these regions are generally under-resourced in terms of mental health services. As a first step to inform service planning and delivery in the English-speaking Caribbean we pooled the available evidence to estimate the prevalence of common mental health problems.

**Methods:**

A comprehensive search of CINAHL, Cochrane Library, EMBASE, MEDLINE, PsycINFO, LILACS, and Web of Science databases, supplemented by grey literature searches was performed until January 2022. Studies conducted in the English-speaking Caribbean that reported prevalence estimates of mental health symptomology or diagnoses in CYP were included. The Freeman-Tukey transformation was applied to calculate the weighted summary prevalence under a random-effects model. Subgroup analyses were also performed to observe emerging patterns in the data. Studies were quality assessed using the Joanna Briggs Institute Prevalence Critical Appraisal Checklist and the GRADE approach. The study protocol was registered with PROSPERO, CRD42021283161.

**Results:**

33 publications from 28 studies representing 65,034 adolescents from 14 countries met the eligibility criteria. Prevalence estimates ranged from 0.8 to 71.9% with most subgroup estimates between 20 and 30%. The overall pooled prevalence of mental health problems was 23.5% (95% CI 0.175–0.302; I^2^ = 99.7%). There was limited evidence of significant variation in prevalence estimates among subgroups. The quality of the body of evidence was judged as moderate.

**Conclusion:**

It is estimated that between 1 in 4 and 1 in 5 adolescents in the English-speaking Caribbean experience symptoms of mental health problems. These findings highlight the importance of sensitisation, screening, and provision of appropriate services. Ongoing research identifying risk factors and validating outcome measures is also needed to inform evidence-based practice.

**Supplementary Information:**

The online version contains supplementary material available at 10.1007/s44192-023-00037-2.

## Introduction

It is estimated that at least 10% of children and young people (CYP) worldwide experience mental health problems with anxiety, depression and behaviour problems appearing to be the most common conditions [1–5]. Despite these estimates the evidence from low- and middle-income countries (LMICs) and developing nations are not well represented in the global pooled prevalence [6]. Yet, a large proportion of CYP below the age of 24 years live in these regions [7, 8].

Research from regions like Sub-Saharan Africa suggested an overall pooled estimate for CYP’s mental health problems at 14.3% rising to 19.8% when using screening questions instead of clinical diagnostic instruments [9]. When focusing on adolescents between ages 10 and 19 years the estimated prevalence was 26.9% with depression being the most prevalent [10]. In India, the prevalence of mental health problems in school aged children was 23.33% [11]. Since 2005, data from Latin America and the Caribbean have also been suggesting that mental health problems comprise around 24% of the health burden, and that at least one in every five CYP experience mental health difficulties [12, 13]. The most recent statistics in 2019 estimated that nearly 16 million adolescents were living with mental health difficulties [14]. Compared to high income countries where mental health problems among CYP ages 4 to 18 years are estimated at 12.7% [15], the estimates from LMICs are sometimes doubled. Experts suggest that mental health in these regions is generally influenced by other public health concerns such as under-nutrition and poverty, as well as other factors like government unrest, lack of education opportunities, gender disadvantage and natural disasters [16–18].

Despite a great need for mental health support, most CYP in LMICs and developing countries do not have access to mental health care [19, 20]. Specifically, the English-speaking Caribbean is considered as underserved and under-resourced in terms of specialist mental health care and evidence-based interventions for CYP and their families [21, 22]. Within the region, some countries are only allocated about 1% to 5% (compared to 8.9% in England) of the health budget towards mental health; of which the majority goes to psychiatric institutions [20, 23]. These statistics are generally representative of both adult and CYP mental health or aggregated with the wider Latin America region, making it difficult to fully understand the scope of the problem for CYP in the English-speaking Caribbean community.

Similar to other regions that are made up of LMICs and among minority ethnic groups, finances, stigma, and lack of awareness alongside social and cultural beliefs also contribute to the major barriers for CYP accessing and receiving mental health support [24, 25]. Together these issues have caused international, regional and local organisations to advocate for appropriate services to promote, protect and care for the mental health of CYP [14, 21, 26, 27]. However, consultations with professional and lived experience experts indicated that an overall understanding of the size and scope of the problem may help decision making around where and how to direct resources [22].

To date, several attempts have been made to highlight the importance of this area and the need for services by providing prevalence data for a range of CYP’s mental health problems. However, the estimates reported in the individual studies have been inconsistent and varied considerably making it difficult to identify a way forward. A recent review highlighted that the prevalence of internalizing behaviour problems ranged from 4.5 to 62%, and 21 to 90.9% for externalizing behaviour problems between 1976 and 2020 [22]. Therefore, there is much to understand by reviewing and pooling the prevalence data at the regional and country level. This approach could give us an oversight of the problem as well as help us compare prevalence rates and perceived performances between countries and thereby learn from countries with best practices and policies.

The English-speaking Caribbean consists of 18 countries or territories [28]: Anguilla, Antigua and Barbuda, The Bahamas, Barbados, Belize, Bermuda, Cayman Islands, Dominica, Grenada, Guyana, Jamaica, Montserrat, Saint Kitts and Nevis, Saint Lucia, Saint Vincent and the Grenadines, Trinidad and Tobago, Turks and Caicos Islands, and the British Virgin Islands. Although the region has similar political, social, educational, and cultural systems, the countries vary in size, geography, socio-demographics and local history [29].

We acknowledge that attempting to estimate the pooled prevalence of mental health problems can be challenging due to the variety of outcome measures used in individual studies as well as the diverse clinical, methodological, and geographic characteristics. However, in an under-resourced region with obvious social disadvantages combined with limited availability and access to CYP mental health services, an estimated prevalence could contribute to service planning and delivery. In addition, the findings could be useful to inform public health and social care priorities. Therefore, this study aimed to critically appraise and synthesize the available evidence on CYP’s mental health in the English-speaking Caribbean to estimate the pooled prevalence of common mental health problems and describe any emerging trends in the data.

## Methods

The review process was conducted following the recommendations from the Joanna Briggs Institute [30, 31] and further informed by the Cochrane handbook [32]. The Preferred Reporting Items for Systematic Reviews and Meta-Analyses (PRISMA) Statement [33, 34] and the Meta-analysis Of Observational Studies in Epidemiology (MOOSE) checklist [35] were also used to guide the reporting of the findings. The protocol was registered with the International Prospective Register of Systematic Reviews (PROSPERO, CRD42021283161) [36]. There were two minor changes to the planned review process as outlined in the protocol. The review team initially planned a secondary investigation to explore the factors associated with mental health in CYP in the English-speaking Caribbean. However, the decision was made to address associating factors in a separate study. This decision was based on the fact that the inclusion criteria for this prevalence review was limited to observational quantitative studies. If we were to investigate factors associated with CYP’s mental health only using this type of study design, our findings could have been biased. Therefore, we will conduct and report findings from that analysis separately, so we can include a review of the broader literature. We were also unable to carry out subgroup analyses based on age. All the identified studies focused on adolescents between 10 and 19 years, and further categorisations (e.g., older versus younger adolescents) were not feasible based on the age ranges reported in the reviewed studies.

### Information sources and search strategy

Electronic academic databases and relevant grey literature sources were initially searched in January 2021 and updated in January 2022. Key search terms relating to “children OR young people” AND “mental health OR well-being” AND “Caribbean” were used in CINAHL, Cochrane Library, EMBASE, MEDLINE, PsycINFO, LILACS, Web of Science, OpenGrey, ResearchGate and the first 10 pages of Google. The reference list of key articles and systematic reviews were also scanned for relevant information. A detailed search strategy was published as part of a scoping review [22].

### Eligibility criteria

The inclusion criteria were defined based on the Condition, Context, Population (CoCoPop) framework [31]; Condition—prevalence of mental health symptoms, context—English-speaking Caribbean, and population of interest—CYP. We excluded studies solely focusing on young people above 18 years and studies when it was unclear if a significant proportion of the sample was under 18 years (e.g. university students). Observational studies (e.g. cohort, case–control, cross-sectional studies, or case series) published as full-text articles were considered for inclusion. We excluded review articles, letters and opinions to the editor, studies evaluating prenatal/parental conditions and studies that did not report sufficient data to estimate mental health prevalence. Studies were also excluded if they primarily examined substance-use disorders, autism spectrum disorders, developmental disorders, intellectual impairment or language/communication disorders as these were beyond the scope of this study. Lastly, to focus on CYP in a general population we also excluded studies solely focusing on young people recruited from health services.

### Study selection process

After duplicates were removed and the eligibility criteria was piloted, the selection process was carried out in three stages by five reviewers (SL, BP, JP, MP, CT). Each record was independently screened by at least two reviewers and involved a third member of the team if unable to reach a consensus. First, titles and abstracts were screened. When both reviewers agreed to “include” or were “unsure” those records were retained for full-text screening. Second, the available full-text articles were read in their entirety and those suitable for the review were selected. Third, an independent researcher (MP), who was not involved in the first two stages of the screening, conducted a 100% verification process to ensure all selected articles met the inclusion criteria. A random sample (66, ~ 25%) of the excluded full text records were also checked for consistency.

### Critical appraisal of the studies

The assessment of the selected studies was carried out using the Joanna Briggs Institute’s critical appraisal tool for assessing the trustworthiness, relevance and results of published prevalence studies [37]. For each of the nine items on this tool, two reviewers judged the criteria as “yes”, “no”, “unclear” or “not applicable”. The items included “Were the study subjects and the setting described in detail?” and “Was the condition measured validly and reliably?”. Validity and reliability were judged as “yes” if the measurement tools used were considered validated or standardised instruments [e.g. Beck Depression Inventory II (BDI-II)] or if details were provided for validity and reliability of the measurement tool in the studied sample. A response of “yes” was also given if the outcomes were assessed based on existing definitions or diagnostic criteria. When disagreements occurred, consensus was achieved through discussions.﻿﻿

The Grading of Recommendations, Assessment, Development and Evaluations (GRADE) approach was then applied to assess the overall quality of evidence [38]. The body of evidence was judged for risk of bias, imprecision, inconsistency, indirectness and publication bias. The GRADE quality appraisal process was also conducted in collaboration with the independent researcher (MP).

### Data extraction

After the extraction sheet was piloted and agreed by the team, at least two reviewers independently extracted the data from each study and the information was then verified by a third reviewer. Data included the article reference, the country and setting where the study was conducted, study design, sample size, presenting problem(s), prevalence data, outcome measures and any other descriptive characteristics of the sample (e.g., sex and age).

### Data analysis

We used the absolute number of observed events and calculated the proportions and 95% confidence intervals (CIs), assuming a binomial distribution. The Freeman-Tukey transformation (arcsine square root transformation) was applied to calculate the average weighted summary prevalence under a random-effects model using the number of events and total sample size [30]. If multiple articles reported findings on the same outcome from the same sample, we only considered data from the manuscript with the most details. Since random effects models give relatively more weight to the results of smaller studies—this was not desirable as smaller studies are typically more prone to bias and of lower quality than larger studies [39]. To account for this, an average weighting process was applied across all studies to control how much information each study provided to the overall analysis when the studies were combined [40]. Given the variation among studies, we stratified the studies to conduct subgroup analyses based on year of study publication, country-specific data, presenting problem(s), sex, recruitment setting(s) and measurement type to investigate if these variables influenced our pooled estimates or contributed to the heterogeneity between studies. These factors were considered important based on evidence from the previous literature [22] and therefore were included as pre-planned analyses in the registered protocol [36].

In distinguishing the subgroups, the studies were grouped by country to allow for inter- and intra-country comparisons. The year of publication was used to observe time trends. We used 10-year periods to coincide with national and regional censuses [41]. The majority of studies did not report separate prevalence estimates by age group or sex, so where possible we used the available data to introduce relevant categories. For example, samples including > 60% females were considered as “majority females”. Based on these unconventional categories and the potential for low power within the subgroups, formal between group statistical significance testing was deemed inappropriate [42, 43]. Instead, we compared CIs of estimates when appropriate and descriptively summarise, subgroup estimates to avoid making misleading recommendations [37].

Average pooled effect sizes were presented using forest plots and 95% confidence intervals (95% CI). I^2^ was used as a measurement for heterogeneity, which was categorized as low (0–40%), substantial (50–90%), or considerable (> 90%). As recommended by Barker et al. [44], statistical tests or “Trim and fill” procedures were not conducted for publication bias as these assumptions may not always be accurate for proportional meta-analyses [45]. Analyses were conducted using Joanna Briggs Institute System for the Unified Management of the Assessment and Review of Information (JBISUMARI) software [46].

## Results

**Fig. 1:**
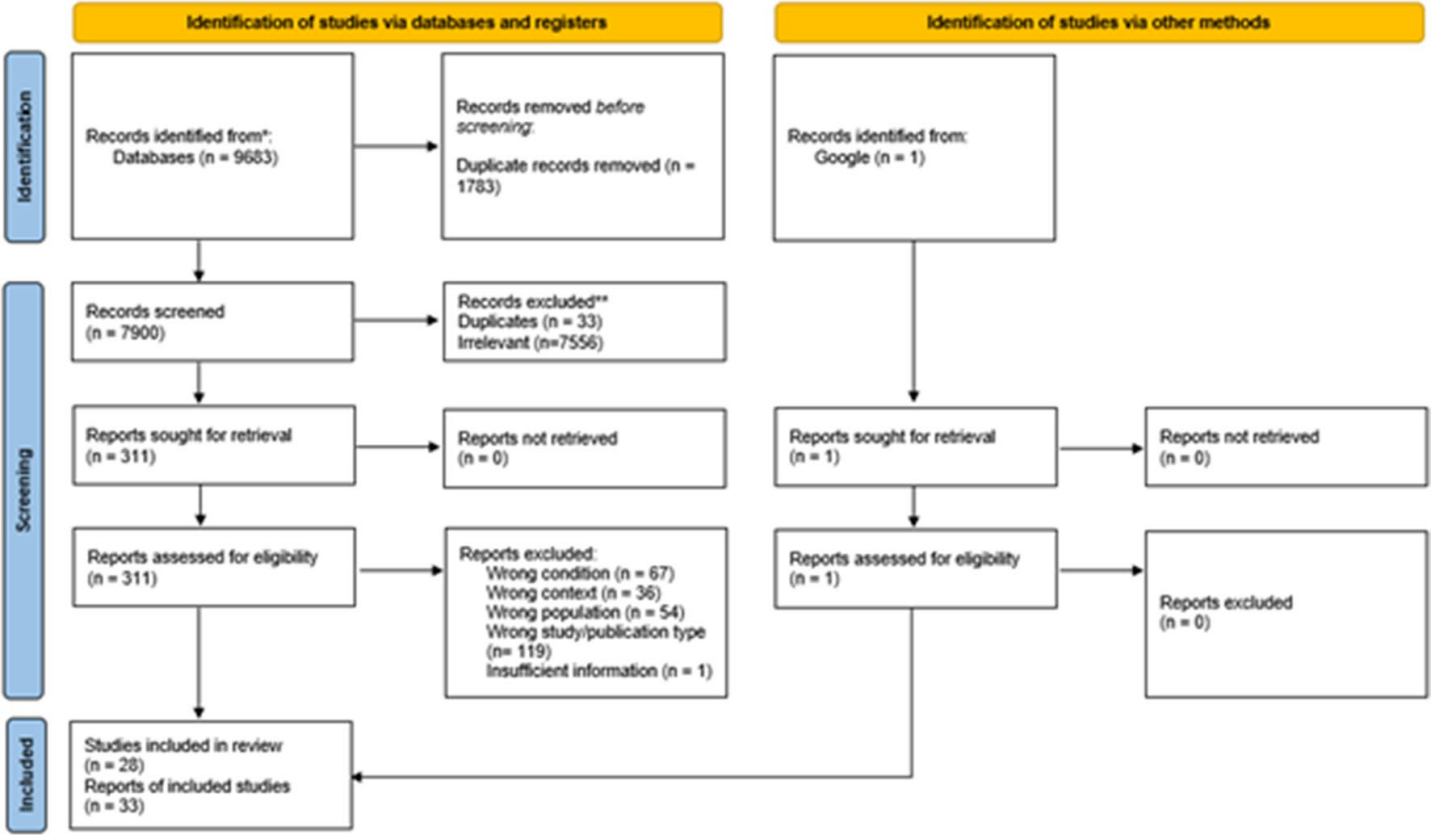
PRISMA flow diagram of the study selection process

The search of the academic databases and grey literature sources resulted in 9684 records (Fig. 1). After duplicates were removed and the titles and abstracts were screened, 311 records were selected for full text assessment. Of these, 278 were excluded based on condition (k = 67), context (k = 36), population (k = 55), study or publication type (k = 119) and insufficient data (k = 1). The remaining 33 articles describing 28 studies and 65,034 adolescents were included in our systematic review and meta-analysis [47–78].

### Characteristics of the included studies

Articles were published between 1997 and 2020 (Table 1). Data from fourteen countries were represented in the reviewed studies. These included Jamaica (k = 10 out of 28, 35.71%), Trinidad and Tobago (k = 8, 28.57%), Guyana (k = 2, 7.14%), The Bahamas (k = 1, 3.57%), Barbados (k = 1, 3.57%), Bermuda (k = 1, 3.57%), St Kitts and Nevis (k = 1, 3.57%), and samples across multiple countries (k = 4, 14.29%, including the British Virgin Islands, Antigua and Barbuda, Cayman Islands, Dominica, Grenada, St Lucia and St. Vincent and the Grenadines). Samples ranged in sizes from 26 to 15,695 and included adolescents between ages 10 and 19 years. Most of the studies (k = 18, 64.29%) were fairly represented in terms of sex (i.e., between 50 and 60% males or females and therefore were considered fairly evenly distributed). Participants were predominantly recruited in education settings (k = 25, 89.29%). Common presenting problems included depressive symptoms (k = 9, 32.14%), suicidality (k = 9, 32.14%), behaviour/conduct problems (k = 3, 10.71%) and disordered eating behaviours and attitudes (k = 4, 14.29%). Three studies (10.71%) focused on general or multiple mental health difficulties including mood and anxiety problems. One study explored depressive symptoms and suicidality in two separate publications [67, 77]. Severity of the problem varied across studies, with some samples reporting mild, moderate and severe symptoms (e.g. [78]). The studies used a variety of outcome measures and scales to assess mental health and behaviour problems including author developed/adapted questionnaires and standardised measures (e.g., Brief Screen for Depression, Beck’s Depression Inventory, Centre for Epidemiology Studies, Eating Attitudes Test, Bulimic Inventory Test, Suicide Behaviour Questionnaire).

**Table 1:** Characteristics of the reviewed studies and the corresponding reports

	Reference: first author, year of publication	Country	Presenting problem explored	Outcome measures	Sample size (N)	MeanAge (SD) or age range in years	Predominant sex (%)	Primary recruitment setting
1	Harrison, 2020	Jamaica	Disordered eating behaviours and attitudes	Eating Attitude Test (EAT)	521	14.8 (not specified)	56.05% females	Education
	Harrison, 2015	Jamaica	Disordered eating behaviours and attitudes	Eating Attitude Test (EAT)	524	14.9 (not specified)	56% females	Education
2	Elledge, 2019	Jamaica	Multiple: suicidality, internal distress (worry, loneliness)	Global School-based Health Survey (GSHS)	1623	11 to 16	51.2% females	Education
3	Oshi, 2018	Barbados	Behaviour and conduct problems	Author developed/adapted questionnaire	8109	14 (3.7)	56.2% females	Education
4	Heron, 2017	Jamaica	Suicidality	Suicidal Behaviours Questionnaire-Revised (SBQ_R)	3471	14.97 (1.7)	58.8% females	Education
5	Kwangu, 2017	Bahamas	Suicidality	Global School-based Health Survey (GSHS)	1357	13 to 17	53.1% females	Education
6	Siziya, 2017a	Guyana	Suicidality	Global School-based Health Survey (GSHS)	2392	Mode 14 to 15	51.5% female	Education
7	Siziya, 2017b	Jamaica	Suicidality	Global School-based Health Survey (GSHS)	1623	Grade 7 to 12	51.4% males	Education
8	Maguire, 2016	Trinidad and Tobago	Behaviour and conduct problems	Trinidad & Tobago Youth Survey (TTYS)	2376	15.4 (not specified)	Nearly 60% females	Education
	Maguire, 2013	Trinidad and Tobago	Behaviour and conduct problems	Trinidad & Tobago Youth Survey (TTYS)	2376	15.4 (not specified)	Nearly 60% females	Education
9	Toussaint, 2015	Trinidad and Tobago	Suicidality	Author developed/adapted questionnaire	4448	18.14 (1.16)	57% females	Education
10	Lowe, 2014	Jamaica, Bahamas, St. Vincent and the Grenadines & St. Kitts & Nevis	Depressive symptoms	Beck Depression Inventory II (BDI-II)	1955	15.3 (0.95)	52.1% females	Education
	Lipps, 2012	Jamaica, Bahamas, St. Vincent and the Grenadines & St. Kitts and Nevis	Depressive symptoms	Beck depression inventory II (BDI-II)	1955	15.5 (0.8)	50.4% females	Education
	Lipps, 2010b	Jamaica, St. Vincent and the Grenadines & St. Kitts and Nevis	Depressive symptoms	Beck depression inventory II (BDI-II)	1738	12 to 19	52% females	Education
11	McFarlane, 2014	Jamaica	Depressive symptoms	The ministry of health screening tool (adaptation of the Diagnostic and Statistical Manual of Mental Disorders 4th Edition)	1312	15 to 19	54.57% females	Community
12	Abdirahman, 2012	Cayman Islands, St Lucia, St Vincent and the Grenadines, and Trinidad and Tobago	Multiple: depression, anxiety, loneliness	Global School-based Health Survey (GSHS)	6780	> 12 to < 16	52.98% females	Education
13	Abel, 2012a	Jamaica	Depressive symptoms	Global School-based Health Survey (GSHS)	3003	12.45 (1.68)	52.65% females	Education
	Abel, 2012b	Jamaica	Suicidality	Global School-based Health Survey (GSHS)	2997	10 to 15	52.69% females	Education
14	Holder-Nevins, 2012	Jamaica	Suicidality	Case notes	26	16 (3.01)	76.9% males	Community Police
15	Lipps, 2010a	Jamaica	Depressive symptoms	Beck Depression Inventory II (BDI-II)	278	15.0 (0.6)	52% females	Education
16	Kukoyi, 2010	Jamaica	Suicidality	Author developed/adapted questionnaire	332	10 to 19	57.3% females	Education
17	Lowe, 2009a	St. Kitts & Nevis	Depressive symptoms	Beck Depression Inventory II (BDI-II)	744	15.5 (0.8)	50.4% females	Education
18	Maharaj, 2008	Trinidad and Tobago	Depressive symptoms	Beck Depression Inventory II (BDI-II)	1290	13 to 19	58.2% females	Education
19	Rudatsikira, 2007	Guyana	Suicidality	Global School-Based Health Survey (GSHS)	1197	14 (not specified)	51% females	Education
20	Ekundayo, 2007	Jamaica	Depressive symptoms	Beck’s Depression inventory II (BDI-II)	748	14 to 19	64.7% females	Education
21	Maharajh, 2006	Trinidad and Tobago	Depressive symptoms	Reynolds Adolescent Depression Scale (RADS)	1845	16.3 (1.13)	60% females	Education
22	Ramberan, 2006	Trinidad and Tobago	Disordered Eating Behaviours and Attitudes	EATing Attitudes Test (EATS-26); Body Shape Questionnaire (BSQ-16); Body Silhouette Chart; Rosenberg Self-Esteem Scale (RSE); Drive for thinness subscale of the Eating Disorder Inventory 2	251	16.3 (1.37)	100% females	Education
23	Marlowe, 2005	Bermuda	Disordered Eating Behaviours and Attitudes	Bulimic Investigatory Test, Edinburgh (BITE); Eating Attitudes Test (EAT-40)	836	12.2 (0.66)	52.5% females	Education
24	Ali, 2004	Trinidad and Tobago	Suicidality	The Suicidal Ideation Questionnaire (SIQ)	1810	16.03 (1.13)	60% females	Education
25	Maharajh, 2004	Trinidad and Tobago	Depressive symptoms	Reynolds Adolescent Depression Scale (RADS); Patient Health Questionnaires (PHQ-9)	198	16.03 (1.13)	60% females	Education
26	Halcón, 2003	Antigua, Barbados, Bahamas, BVI, Dominica, Grenada, Guyana, St. Lucia	Multiple	Author developed/adapted questionnaire	15,695	10 to 18	61% females	Education
27	Bhugra, 2003	Trinidad and Tobago, Barbados	Disordered Eating Behaviours and Attitudes	Bulimia Investigatory Test, Edinburgh (BITE); DSM-III-R Bulimia Diagnostic Interview	362	14.9 (1.17)	100% females	Education
28	Deosaran, 1997	Trinidad and Tobago	Behaviour and conduct problems	Author developed/adapted questionnaire	486	< 18	75.31% males	Community Group Home

### Quality of the included studies

**Fig. 2:**
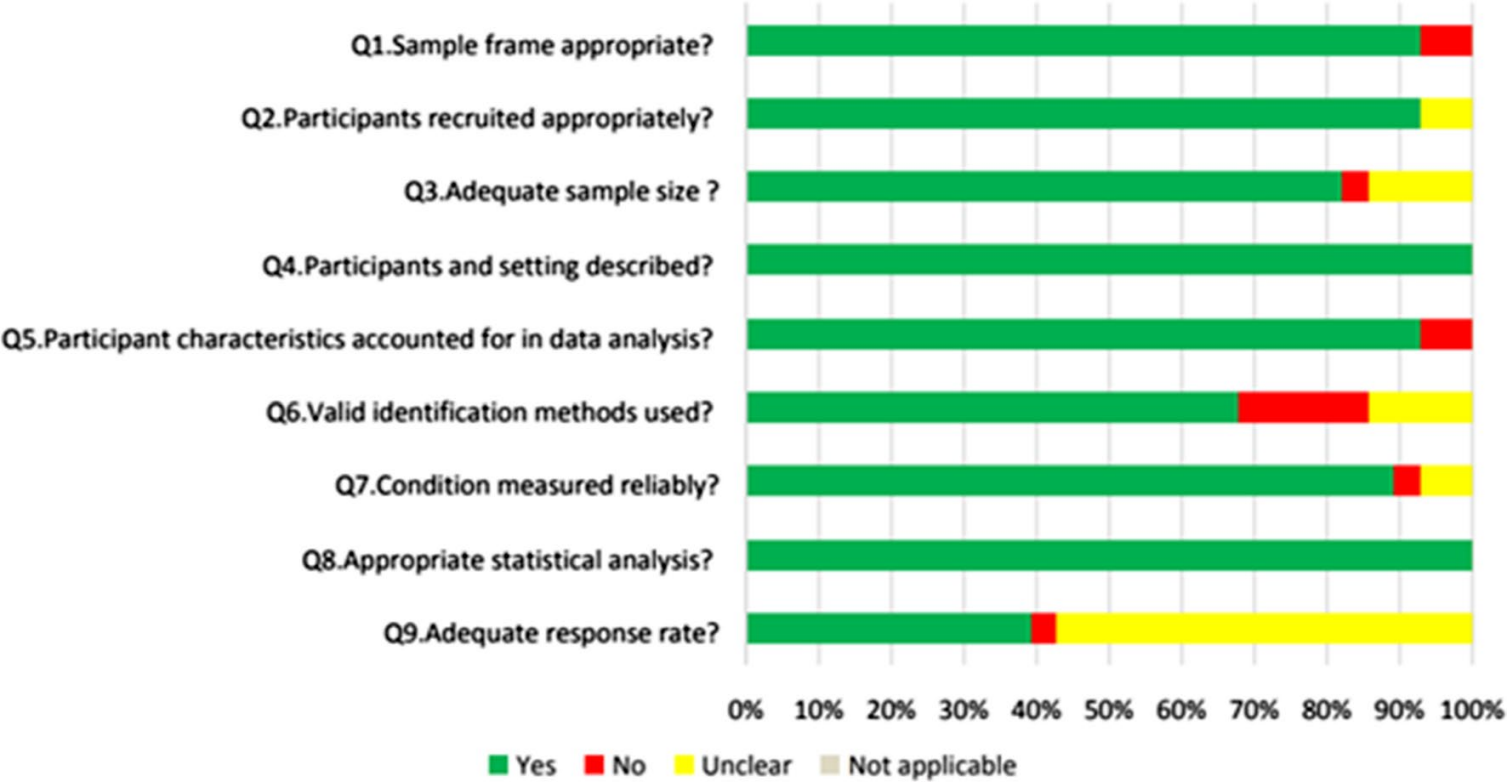
Quality assessment of the reviewed studies

Methodological quality varied across the included studies (Supplementary Information 1). Only 8 out of the 28 studies (28.57%) were judged as “yes” on all nine of the criteria. The remaining studies were judged as having limitations in at least one criterion. Two studies (7.14%) were judged as meeting less than half of the criteria. Most studies (k = 26, 92.86%) described a process that appropriately recruited the targeted sample. All studies clearly described the study participants and the setting as well as conducted appropriate statistical analyses. Most studies (k = 26, 92.86%) accounted for participant characteristics in the data analysis. Regarding outcome measurements, nineteen studies (67.86%) used valid identification methods and most studies (k = 25, 89.29%) collected data in a consistent and reliable way. Twenty-three (82.14%) studies reported adequate sample sizes while some studies (k = 4, 14.29%) were unclear on how the sample sizes were determined. Only 11 studies (35.48%) were considered to have an adequate response rate of more than 70%. Figure 2 provides an overview of the quality assessment.

The overall quality of the available evidence was judged as moderate according to GRADE. The body of evidence was rated upwards for precision because most of the studies included large samples (> 300 participants) and reported many events. Based on the nature of the included studies publication bias was not suspected [44]. Our rating was downgraded mainly because most of the included studies had at least one limitation which could introduce some risk of bias. There was also some variability in the prevalence estimates of included studies, and only limited representation from the wider community settings.

### Prevalence of mental health and behaviour problems

**Fig. 3:**
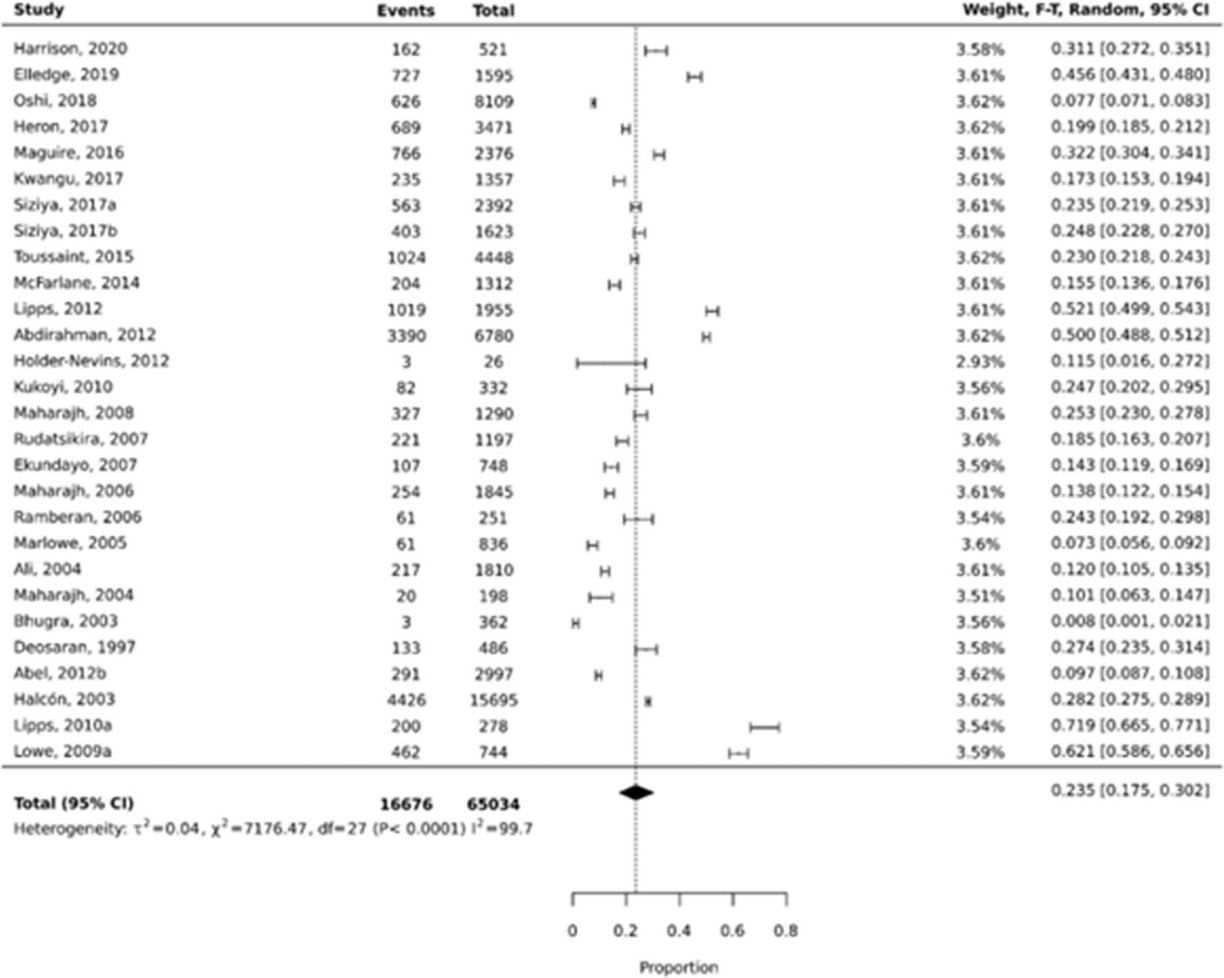
Overall pooled prevalence for mental health and behaviour problems

No obvious outliers (defined as not overlapping with any of the nearest data points) were identified, therefore all studies were included in the meta-analysis. The overall weighted pooled prevalence of mental health and behaviour problems among adolescents was 23.5% (95% CI 0.175–0.302; I^2^ = 99.7%) (Fig. 3). Prevalence estimates ranged from 0.8% (95% CI 0.001–0.021) in a study that examined disordered eating behaviours in a sample of female adolescents [47] to 71.9% (95% CI 0.665–0.771) in a Jamaican study that explored depressive symptoms [57].

### Subgroup prevalence estimates

Based on observations, most of the subgroup prevalence estimates were between 20 and 30% and fewer estimates were between 10 and 20%. Single studies conducted in Barbados and Bermuda and pooled estimates for studies exploring disordered eating/body image issues had estimates less than 10%. A single study conducted in St. Kitts and Nevis exploring depressive symptoms and pooled estimates for studies exploring general or multiple mental health problems had higher estimates above 30%. However, the CIs were wide within groups and there was some overlap between groups so statistical significance of the difference between the groups could not be determined with certainty. Substantial and considerable amounts of heterogeneity was also observed among the included studies. Table 2 provides further details of the estimates for the different subgroups.

**Table 2:** Prevalence estimates based on predefined groups

Subgroup	# of reports	N participants	n events	Pooled estimate (%)	95% Confidence intervals	I^2^ (%)
Publication year						
1990–1999	1	486	133	27.4	0.235–0.314	0
2000–2009	11	24,941	6105	15.4	0.074–0.257	99.6
2010–2021	16	39,569	10,227	27.1	0.186–0.366	99.7
Country						
Jamaica	10	12,909	2709	24.9	0.139–0.378	99.6
Trinidad and Tobago	8	12,669	2744	17.1	0.103–0.406	99.2
Guyana	2	3589	782	20.9	0.162–0.262	92
Bahamas	1	1348	235	17.4	0.155–0.195	0
Barbados	1	8109	626	7.7	0.071–0.083	0
Bermuda	1	836	61	7.3	0.056–0.092	0
St Kitts and Nevis	1	744	462	62	0.586–0.656	0
Multiple	4	24,792	8838	28.5	0.057–0.599	99.9
Presenting problem(s)						
Behaviour/conduct	3	10,971	1525	21.2	0.077–0.390	99.6
Depressive symptoms	9	11,338	2724	27.4	0.128–0.452	99.7
Eating/body image	4	1970	232	7.6	0.003–0.227	98.9
Suicidality	10	19,644	3724	18.5	0.150–0.223	97.5
General/multiple	3	24,070	8543	41.0	0.282–0.545	99.7
Outcome measures						
Standardised	22	35,938	10,321	23.1	0.155–0.318	99.7
Author developed/adapted	5	29,070	6290	21.4	0.137–0.304	99.6
Case notes	1	26	3	11.5	0.016–0.272	0
Recruitment settings						
Education	25	63,210	16,276	23.0	0.161–0.306	99.8
Community	3	1824	339	18.9	0.107–0.288	92.4
Sex						
Majority females	8	23,285	5798	12.4	0.056–0.213	99.5
Majority males	2	514	136	20.2	0.071–0.376	76.1
Fairly evenly distributed	18	41,237	10,681	27.9	0.196–0.370	99.7

## Discussion

### Summary of the findings

To the best of our knowledge this is the first study to estimate the regional and country-level pooled prevalence of adolescent mental health in the English-speaking Caribbean. We appraised and synthesised the evidence from 28 studies (33 articles) published between 1997 and 2020, resulting in an overall pooled prevalence of 23.5%. In general prevalence rates appeared to be relatively high in the last 23 years. Taking into account the overall pooled prevalence alongside subgroup prevalence data we estimated that around one in every four or five adolescents in the region may experience mild to severe mental health or behaviour problems, including depressive symptoms and suicidality during adolescence.

### Interpretation of the findings

The overall prevalence estimate appears to be higher than some global estimates [2, 3] and high-income countries [15] but comparable to those in similar regions [10, 11]. Our findings also confirm what was estimated for the wider Latin America and the Caribbean in 2005 [12] and 2019 [14]. Therefore, the current findings further highlight the importance of regional as well as national attention to CYP’s mental health as outlined by UNICEF and Healthy Caribbean Youth [14, 27]. It is also possible that the high estimates for specific presenting problems like depressive symptoms and behaviour/conduct problems can be attributed to the social disadvantages experienced by CYP in the region [79]. Previous research identified teenage pregnancy and witnessing or participating in violence as being common among CYP from this region [80]. Therefore, the findings from this review are not surprising as there is a wealth of evidence suggesting that these factors are associated with poorer mental health and wellbeing in CYP [81].

Even within the region individuals from different cultures and communities may express and interpret mental health problems differently [82]. Similarly, mental health symptoms may present differently across genders and age groups but most of the reviewed studies rarely provided separate prevalence data which limited the scope of our analysis. In our attempts to explore subgroup differences we observed overlapping CIs, so it is possible that prevalence rates were similar irrespective of study and participant characteristics. In addition, for some categories, the CIs were very wide and there were fewer studies or smaller sample sizes which lowered our levels of certainty [42, 43]. Despite these statistical assumptions, most of the subgroup estimates were considerably high (> 20%). In areas where lower estimates were observed, those were single studies or explored disordered eating behaviours. This is consistent with the global evidence suggesting that currently eating disorders are generally lower in low- and middle-income and developing countries [83].

Notably, a larger number of included studies (k = 18) were from Jamaica and Trinidad and Tobago, indicating more research activity in comparison to the other countries. We also observed consistently high estimates over the last 23 years but with further investigations a different message might be obtained for the different countries. In line with this, several studies highlighted the high prevalence of suicidality and depressive symptoms. Acknowledging that some studies were conducted more than 5 years ago so may not reflect current burden, this may still require urgent attention. Similarly, although fewer studies focused on behaviour/conduct problems and eating/body images issues, the presentations appeared to be extreme compared to other countries. For example, young people are more likely to engage in offending criminal behaviours [84] and extreme weight-control behaviours. [85] These are important findings that can be used to better understand the landscape of CYP’s mental health and wellbeing in the Caribbean. It is possible that any mental health and wellbeing policies and practices implemented until 2020 may need to be reviewed, monitored and evaluated. This review also confirms the need for more trained mental health practitioners [86] and both short- and long-term community-based support services and programmes [87].

Major life changing events like natural disasters and changes in governments would have occurred over the last 30 years that could have affected each country differently. Similarly, there is global evidence suggesting that CYP’s mental health was negatively affected by the Covid-19 pandemic [88]. The reviewed studies were conducted before the start of the pandemic and therefore could form a baseline to inform any new emerging evidence on CYP’s mental health and wellbeing in the Caribbean.

### Implications for service planning and delivery

It is apparent that a significant number of adolescents experience poor mental health that could present challenges to their wellbeing. Based on the current findings, with most of the data being collected from education settings, a public health approach may be needed to help address adolescent mental health and wellbeing. Therefore, adolescents, their families and schools may need to be sensitised on the importance of mental health and wellbeing alongside being made aware of mental ill-health. Consequently, if embedded within education policies, systematic and ongoing screening could make it possible to identify symptoms of mental health and behaviour problems and provide an opportunity for the provision of early interventions. The high percentage of CYP in the general population experiencing mental health problems also highlights a need for more research to identify the pathways to accessing services to fully understand how much the level of severity impacts decisions to access care. This is an important next step if we are to consider implementing early interventions. With this approach early interventions can be embedded as part of the curriculum and whole school approaches can be adopted. It is well established that early identification and intervention for mental health and behaviour problems in childhood have the potential to improve short-and medium-term outcomes as well as prevent negative outcomes in adulthood [89–91]. There is also growing evidence that whole school approaches can be effective [92] and easily implemented [93] to support CYP’s mental health.

However, to effectively implement these recommendations, there are some important questions that should be addressed. First, what are the risk and protective factors associated with CYP’s mental health in the Caribbean region? A review bringing together the evidence on these factors could provide a knowledge base to inform interventions and targeted services to meet the needs of all CYP. Second, how best to measure symptoms of mental health and behaviour problems in this region? Although, the CIs of estimates derived from standardised measures and author developed/adapted measures in our meta-analyses overlapped, our findings would suggest the need for future studies using a variety of instruments so they can be validated. By doing this we may also obtain more updated evidence regarding the current state of CYP's mental health since the start of the Covid-19 pandemic. This is also necessary to address the dearth of evidence on younger children (< 10 years). Third, what are the barriers and facilitators to accessing and receiving mental health support for CYP? An understanding of this is essential to further inform a clear pathway to services. Fourth, what are the challenges and opportunities for carrying out CYP mental health research activity in the Caribbean? And fifth, what are the barriers to the application of the research findings? Owing to the limited evidence in some areas of CYP's mental health, ongoing research will be needed. Therefore, it will be useful to determine the needs of researchers and practitioners to provide relevant training and resources.

### Strengths and limitations

The main strength of this review is that being the first study to estimate the pooled prevalence of adolescent mental health and behaviour problems in the English-speaking Caribbean region, we were able to synthesise a wealth of evidence that can be used to inform service planning and delivery. We also conducted subgroup analyses which revealed country-specific and population-specific data that can be used to inform targeted programmes. If our recommendations are considered, this can impact the lives of many CYP. Another strength is the large dataset. We pooled data from 28 studies representing 65,034 adolescents across 14 countries which could strengthen the validity and reliability of our findings. We also adopted several measures (e.g., rigorous inclusion/exclusion criteria, search terms and multiple reviewers) to avoid missing relevant studies and to control for bias and misinformation while adhering to established guidelines for prevalence reviews.

Despite these efforts some limitations must be acknowledged. The data from a variety of sources were pooled in this study so caution should be taken when interpreting the findings. Comparability of the included studies was complicated by the diversity of recruitment methods, demographics of participants, assessment tools, analytic strategies, and reporting standards. In addition, the approach adopted for the proportional meta-analysis allowed each study to contribute almost equally to the overall pooled average. Despite some experts suggesting the strengths of this approach to control for biases and insufficient power of individual studies, we acknowledge that the individual studies varied in sample sizes and events. However, not all outcome measures were validated and/or standardised for the studied population so applying varying weights could have introduced further bias. To further understand the heterogeneity among the studies, we expect that further research could modify our approach to subgroup analyses to increase precision and account for severity of symptoms. Although our subgroup analyses were pre-planned, our analyses are observational by nature and are not based on randomized comparisons. Nonetheless the high heterogeneity observed among the included studies appear to be common in these types of reviews and expected because of the clinical and methodological diversity of the included studies [94]. Data from some countries were also under-represented in our dataset. Therefore, caution should be taken if any attempts are made to generalise our findings across the different countries. If this is done, it is possible that overestimation or underestimation bias may occur. Lastly, the quality of the included evidence was judged as moderate which could mean that the true prevalence could be slightly different from our estimates.

## Conclusion

Mental health and behaviour problems affect a large proportion of adolescents in the English-speaking Caribbean. Overall and subgroup prevalence estimates were mainly between 20 and 30%. There was limited evidence of significant variation in prevalence estimates over time and between countries, presenting problems, sex or types of outcome measures used. Systematic screening, sensitisation, and ongoing support for adolescents’ mental health and wellbeing in schools and other education settings are recommended. Additional studies are needed to explore risk and protective factors associated with mental health, as well as to validate additional outcome measures to assess CYP mental health and wellbeing in the region.

## Supplementary Information

Below is the link to the electronic supplementary material.

Supplementary file1 (PDF 48 KB)

## Data Availability

The data and materials associated with this manuscript are included in the manuscript.

## Abbreviations

CYP: Children and young people
LMIC: Low- and middle-income country
CI: Confidence interval
