# Supplementary material for: Prevalence of mental health and behaviour problems among adolescents in the English-speaking Caribbean: systematic review and meta-analysis
Source: Discov Ment Health. 2023 May 18;3(1):11. doi: 10.1007/s44192-023-00037-2 (PMC10196301; doi:10.1007/s44192-023-00037-2)
Supplement: Supplementary file 1 — Supplementary file1 (PDF 48 KB) [file 44192_2023_37_MOESM1_ESM.pdf]

	Study reference(s)	Quality appraisal items*									Total # of YES
		1	2	3	4	5	6	7	8	9	
1	Halcón, 2003	YES	YES	YES	YES	YES	NO	NO	YES	Unclear	6
2	Kwangu, 2017	YES	YES	YES	YES	YES	YES	YES	YES	YES	9
3	Oshi, 2018	YES	YES	YES	YES	YES	NO	YES	YES	Unclear	7
5	Marlowe, 2005	YES	YES	YES	YES	YES	YES	YES	YES	YES	9
6	Abdirahman, 2012	YES	YES	YES	YES	YES	YES	YES	YES	Unclear	8
8	Rudatsikira, 2007	YES	YES	YES	YES	YES	YES	YES	YES	YES	9
9	Siziya, 2017a	YES	YES	YES	YES	YES	YES	YES	YES	YES	9
10	Abel, 2012a; Abel, 2012b	YES	YES	YES	YES	YES	YES	YES	YES	Unclear	8
11	Ekundayo, 2007	YES	YES	YES	YES	NO	YES	YES	YES	Unclear	7
12	Elledge, 2019	YES	YES	YES	YES	YES	YES	YES	YES	Unclear	8
15	Harrison, 2015; Harrison, 2020	YES	YES	YES	YES	YES	YES	YES	YES	NO	8
16	Heron, 2017	YES	YES	YES	YES	YES	YES	YES	YES	YES	9
18	Holder-Nevins, 2012	NO	Unclear	NO	YES	NO	Unclear	Unclear	YES	Unclear	2
19	Kukoyi, 2010	YES	YES	YES	YES	YES	NO	YES	YES	Unclear	7
21	Lipps, 2010a	YES	YES	Unclear	YES	YES	YES	YES	YES	Unclear	7
24	McFarlane, 2014	YES	YES	YES	YES	YES	Unclear	YES	YES	YES	8
26	Siziya, 2017b	YES	YES	YES	YES	YES	NO	YES	YES	YES	8
28	Lipps, 2012; Lowe, 2014; Lipps, 2010b	YES	YES	YES	YES	YES	YES	YES	YES	YES	9
29	Lowe, 2009a	YES	YES	YES	YES	YES	YES	YES	YES	YES	9
31	Ali, 2004	YES	YES	YES	YES	YES	YES	YES	YES	Unclear	8
33	Deosaran, 1997	YES	Unclear	Unclear	YES	YES	Unclear	Unclear	YES	Unclear	4
34	Maguire, 2016; Maguire, 2013	NO	YES	YES	YES	YES	Unclear	YES	YES	Unclear	6
35	Maharaj, 2008	YES	YES	YES	YES	YES	YES	YES	YES	YES	9
37	Maharajh, 2006	YES	YES	YES	YES	YES	YES	YES	YES	Unclear	8
38	Ramberan, 2006	YES	YES	YES	YES	YES	YES	YES	YES	Unclear	8
39	Toussaint, 2015	YES	YES	YES	YES	YES	NO	YES	YES	Unclear	7
41	Maharajh, 2004	YES	YES	Unclear	YES	YES	YES	YES	YES	Unclear	7
42	Bhugra, 2003	YES	YES	Unclear	YES	YES	YES	YES	YES	YES	8

*Quality appraisal items

1 - Sample frame appropriate?
2 - Participants recruited appropriately?
3 - Adequate sample size?
4 - Participants and setting described?
5 - Participant characteristics accounted for in data analysis?
6 - Valid identification methods used?
7 - Condition measured reliably?
8 - Appropriate statistical analysis?
9 - Adequate response?
